# Supplementary material for: The interactome of intact mitochondria by cross-linking mass spectrometry provides evidence for coexisting respiratory supercomplexes
Source: Mol Cell Proteomics. 2017 Dec 8;17(2):216–32. doi: 10.1074/mcp.RA117.000470 (PMC5795388; doi:10.1074/mcp.RA117.000470)
Supplement: Supplemental Data [file supp_RA117.000470_133922_0_supp_23978_fzffwf.zip › spectra_annotation/mito_spectra_annotation/188-2-5-1-2-1.pdf]

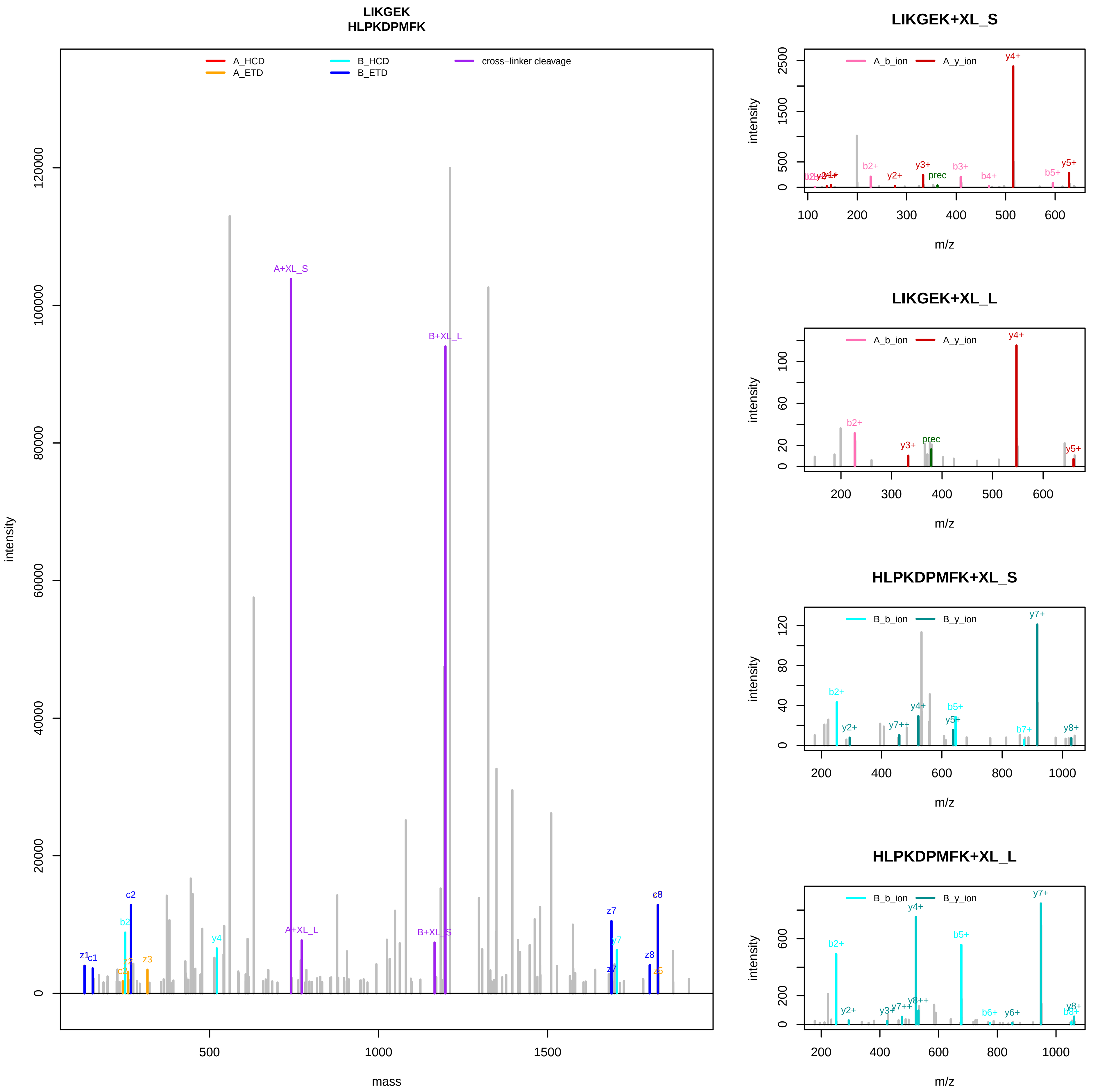

LIKGEK
HLPKDPMFK
A_HCD
A_ETD
B_HCD
B_ETD
cross−linker cleavage
intensity
mass
A+XL_S
B+XL_L
A+XL_L
B+XL_S
z1
c1
c2
b2
z2
c2
z3
y4
z7
y7
z7
z8
c8
z5
LIKGEK+XL_S
LIKGEK+XL_L
HLPKDPMFK+XL_S
HLPKDPMFK+XL_L
A_b_ion
A_y_ion
B_b_ion
B_y_ion
m/z
y4+
y5+
b5+
b4+
b3+
y3+
prec
y2+
b2+
b1+
y7+
y8+
b7+
b6+
y6+
b8+
y7++
y8++
y3+
